# Impact of Thermal Processing and Carbohydrate Carriers on Amino Acids, Sugars, Phenolic Compounds, and Maillard Reaction Markers Relevant to Nutritional Quality and Safety of *Rosa canina* L. Juice Powders

**DOI:** 10.3390/molecules30183805

**Published:** 2025-09-18

**Authors:** Anna Michalska-Ciechanowska, Jessica Brzezowska, Maria Dolores del Castillo, Marta Beatriz López-Parra, Lidia Montero, Nancy Nicolet, Igor P. Turkiewicz, Wilfried Andlauer

**Affiliations:** 1 Department of Fruit, Vegetable and Plant Nutraceutical Technology, Faculty of Biotechnology and Food Science, Wrocław University of Environmental and Life Sciences, 51-630 Wrocław, Poland; jessica.brzezowska@upwr.edu.pl (J.B.); igor.turkiewicz@upwr.edu.pl (I.P.T.); 2 Instituto de Investigación en Ciencias de la Alimentación, Consejo Superior de Investigaciones Científicas (CSIC)-Universidad Autónoma de Madrid (UAM), 28049 Madrid, Spain; mdolores.delcastillo@csic.es (M.D.d.C.); martab.lopez.parra@csic.es (M.B.L.-P.); 3 Laboratory of Foodomics, Institute of Food Science Research, CIAL, CSIC, Nicolás Cabrera 9, 28049 Madrid, Spain; lidia.montero@csic.es; 4 Institute of Life Sciences, School of Engineering, University of Applied Sciences and Arts Western Switzerland (HES-SO Valais Wallis), Rue de l’Industrie 19, 1950 Sion, Switzerland; nancy.nicolet@hevs.ch

**Keywords:** *Rosa canina* L. powders, carriers, thermal processing, process-related molecules, non-enzymatic glycation, food safety and quality, antioxidant molecules, risk-benefit balance

## Abstract

*Rosa canina* L. powders are rich in bioactive compounds; however, thermal processing and carrier addition can markedly alter their composition, affecting nutritional quality, safety and potential health effects. This study assessed the impact of juice pasteurization, followed by freeze-drying and spray drying with maltodextrin, inulin, trehalose, or palatinose, on amino acids, sugars, phenolic compounds, and Maillard reaction products. In carrier-added samples, total amino acid content ranged from 364.6 to 430.4 mg/100 g dry matter (DM). Tryptophan and γ-aminobutyric acid (GABA) were proposed as indicator compounds, with their distinct stability patterns under pasteurization and drying providing a reliable measure of processing effects while representing both essential (tryptophan) and non-essential (GABA) amino acids of nutritional relevance. Carriers reduced total sugar content ≈ 2.5-fold (from 16.7 to 6.7 g/100 g powder DM) but modified sugar profiles. Powders obtained from pasteurized juice with inulin showed the highest level of free amino groups (0.49 g N-α-acetyl-*L*-lysine/100 g powder DM), the lowest fructosamine content (44.8 mmol DMF/100 g powder DM), and total phenolics of 10.4 g GAE/100 g powder DM. Fructosamine, an early Maillard reaction marker, proved valuable for predicting powder quality, with lower levels linked to pasteurization and carriers with low glycation potential. Acrylamide was absent in all samples (LC–MS/MS), confirming chemical safety. Processing parameters and carrier type were key determinants of nutritional composition and safety, with inulin-based powders from pasteurized juice offering a balanced profile of free amino acids and phenolics, while minimizing fructosamine formation. The combined use of amino acid profiling, phenolic quantification, and early Maillard reaction markers provides a powerful approach for quality evaluation of fruit-based powders within glycoscience.

## 1. Introduction

The growing interest in plant-based products, rich in bioactive compounds with health-promoting properties, has intensified research into effective processing methods. Among these, drying is widely employed to preserve plant matrices and retain their bioactives, while facilitating their incorporation into various food systems. In this context, plant powders are gaining increasing attention as convenient and versatile food ingredients due to their potential to enhance the nutritional and functional properties of numerous food products [1]. Powders offer an alternative to seasonal raw foods. They also contribute to shelf-life extension and broaden the functional applications of a wide range of fruits, vegetables, and herbs [1], especially when produced from juices and/or extracts that enable reconstitution into soluble forms. However, obtaining powders from liquids (juices, extracts, etc.) with a bioactives profile comparable to that of the fresh counterparts requires controlled processing conditions. This comprises all stages of production, including raw material pretreatment and the formulation of the liquid feed. Beyond the challenge of preserving bioactives, increasing attention is being directed toward to the potential formation of process-induced contaminants [2,3]. This concern arises particularly in the context of the structural complexity and diversity of plant matrices [4].

**Figure 1:**
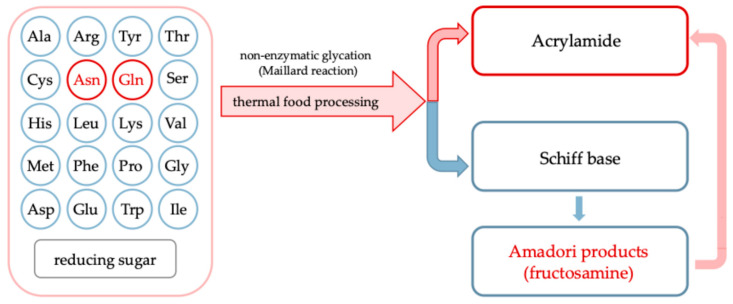
Pathways of amino acid involvement in the non-enzymatic glycation (Maillard reaction) under heat treatment of foods.

During processing, especially under thermal treatment, undesirable compounds can be formed through complex chemical transformations, such as non-enzymatic glycation also called Maillard reaction, involving endogenous plant constituents and exogenous additives [4,5,6]. Carriers, which are commonly used to improve the drying behavior and stability of plant-based liquid feeds, can also significantly influence the formation of process contaminants [7]. Their chemical composition and interactions with matrix components can promote or inhibit non-enzymatic glycation and caramelization reactions leading to formation of acrylamide, hydroxymethyl-*L*-furfural and other products comprising food safety. Figure 1 illustrates in a simplified way the Maillard reaction between free amino acids and reducing sugars in food quality and safety [2]. Asparagine (highlighted in red) leads to acrylamide formation *via* a specific decarboxylation and deamination pathway, while other amino acids (in blue) primarily contribute to the formation of Amadori products, such as fructosamine, detectable by the nitro blue tetrazolium (NBT) assay. The relative contribution of each amino acid depends on its nucleophilicity and abundance in the matrix. These steps lead to the formation of molecules considered chemical indicators for assessing the risk-benefit balance of non-enzymatic glycation during thermal food processing [8].

Therefore, the design of processing steps should be carefully adjusted to ensure the preservation of the nutritional and functional qualities of the plant powders while minimizing the formation of potentially harmful molecules in final products.

*Rosa canina* L. is an underutilized wild-growing shrub whose red fruits, known as rosehips, are recognized as an exceptional source of vitamin C, polyphenols, carotenoids, and other antioxidants contributing to their strong health-promoting potential [9,10]. Despite these nutritional benefits, their astringent taste and fibrous texture limit direct consumption and culinary appeal. Moreover, processing of this raw material presents additional challenges due to its relatively high content of sugars and organic acids, which can promote undesirable chemical reactions during thermal treatment and drying. Converting *Rosa canina* L. into a powder form offers a promising solution by improving sensory attributes and enhancing applicability in food systems as a valuable functional ingredient [11]. The transformation of *Rosa canina* L. liquid matrices into powder through processes like juicing, pasteurization, and carrier-assisted drying induces numerous chemical alterations. These changes include the degradation and interactions of native bioactives, as well as the formation of new compounds, some of which may exert beneficial effects, while others may pose potential health risks. Particular attention should be given to above mentioned process contaminants such as non-enzymatic glycation products or furans, whose presence and concentrations depend on processing conditions and the type of carrier used. Carbohydrate-based carriers, commonly added to improve drying efficiency, may interact with sugars, organic acids, and bioactive compounds, thus influencing the nutritional quality and safety of the final product.

Considering the above, the present study aimed to evaluate the impact of juicing (in combination with pasteurization) and subsequent powdering (through carrier-assisted drying employing carbohydrate-based carriers) on the degree of processing in *Rosa canina*-based powders. This study assessed the impact of juice pasteurization, followed by freeze-drying or spray drying with maltodextrin, inulin, trehalose, or palatinose, on amino acids, sugars, phenolic compounds, and Maillard reaction products to determine the nutritional quality and food safety of plant-derived powdered products for establishing the nutritional risk-benefit balance of food processing.

## 2. Results

### 2.1. Effect of Pasteurization on Rosa canina L. Juice Composition

The amino acids content in freeze-dried juice of *Rosa canina* L. averaged 0.87 g/100 g powder dry matter (DM) for both pasteurized and non-pasteurized samples, which is considerably lower than levels reported for whole fruits of *Rosa roxburghii* and *Rosa sterilis* [12]. This difference can be attributed to the specific initial composition of the variety as well as the losses occurring during the juicing process.

The effect of pasteurization on the amino acid profile of *Rosa canina* L. juice was evaluated by comparing freeze-dried samples obtained from both pasteurized and non-pasteurized juices. In this case, no carriers were used during freeze-drying to avoid their influence on composition changes. As shown in Table 1, pasteurization induced statistically significant changes in the total content of amino acids, including both essential and non-essential amino acids (Supplementary Data S1). The findings indicated that pasteurization, while generally preserving the amino acid profile, may cause changes in their concentration in *Rosa canina* L. juice, indicating that thermal processing can influence amino acids as previously observed for selected non-essential amino acids in watermelon juice [13], potentially due to Maillard-type reaction.

No statistically significant differences were observed between non-pasteurized and pasteurized samples regarding sugars content, free amino groups analyzed by OPA assay and total phenolics content (Table 1). Since thermal processing promotes the formation of various compounds through non-enzymatic glycation reactions [14], fructosamine has been identified in foods, where it contributes to the development of characteristic aroma, flavor, and color in thermally processed or dehydrated products [15]. In this study, fructosamine levels were approximately 24% higher in the non-pasteurized juice than in the pasteurized one, suggesting that thermal treatment may trigger the progression of the Maillard reaction and promote conversion of fructosamine into more advanced glycation end-products (AGEs), thus reducing its detectable concentration in the assay. This pattern aligns with the recognized instability of Amadori intermediates under heat, which undergo degradation and transformation into dicarbonyl compounds, i.e., precursors to AGEs [16]. To confirm this hypothesis, further analyses are required, specifically targeting the detection of intermediate Maillard reaction products, dicarbonyls (e.g., glyoxal, methylglyoxal), and direct AGEs (e.g., carboxymethyl-*L*-lysine, etc.). Such insight would clarify whether pasteurization primarily induces degradation of fructosamine, facilitates its transformation into AGEs, or alters its measurable recovery due to matrix interactions. Understanding these pathways is crucial for evaluating the nutritional and functional implications of thermal treatment in fruit-based food systems.

### 2.2. Effect of Drying (Powdering) on Rosa canina L. Powders Composition

#### 2.2.1. Amino Acids in Powders

In total, 17 amino acids were identified and quantified in the analyzed powders, including nine essential and eight non-essential amino acids (Supplementary Data S1) [17]. These compounds were detected both in the control samples (without carrier addition) and in the samples containing carriers. In most cases, the carrier addition prior drying reduced the total amino acid content in the powders. In products obtained from non-pasteurized juice, the total amino acid content decreased by approximately 45%, whereas in those obtained from pasteurized juice, the reduction was around 22% compared to freeze-dried controls. The difference can be attributed to the initial amino acids content in pre-treated juices. In the carrier-added samples, essential amino acids accounted for 21% of the total amino acid content, while non-essential amino acids constituted the remaining 79%. This distribution pattern is consistent with that reported in *Rosa roxburghii* and *Rosa sterilis*, where non-essential amino acids also predominate [12]. Among the essential amino acids, histidine was dominant, regardless of the carrier type or the drying technique applied. Histidine accounted, on average, for 55.6% of the total essential amino acids, followed by tryptophan (20.3%), leucine (7.1%), phenylalanine (5.1%) and threonine (4.9%). The remaining essential amino acids, including methionine, valine, isoleucine and lysine, each contributed less than 3% to the total essential amino acid pool. Overall, when considering the sum of amino acids, the drying technique applied had no significant effect on their profile, suggesting that spray drying, as a more economical and efficient technique [18], may be recommended for powder production from nutritional perspective. Similarly, the type of carrier used did not substantially differentiate the total amino acids content in the powders; however, in case of inulin-added products the content of lysine, a highly reactive amino acids, was higher in non-pasteurized inulin-added products, further indicating that the observed differences in particular components were not attributed only to the drying technique.

Among the non-essential amino acids, arginine was the predominant compound, accounting on average for 66% of the total identified non-essential amino acids, followed by glutamic and aspartic acids. A similar profile of dominant non-essential amino acids has been reported for *Rosa roxburghii* and *Rosa sterilis*, where non-essential amino acids also prevailed [12]. As observed for essential amino acids, neither the drying technique nor the type of carrier had a significant effect on their content in the analyzed powders.

Although asparagine and glutamine have previously been reported in *Rosa canina* L. fruits and juices [17], neither of these amino acids (nor acrylamide; discussed below) were detected in the powdered products analyzed in this study. This finding is particularly noteworthy as asparagine is the main precursor of acrylamide formed *via* heat-induced Maillard-type pathways [2] (Figure 1). In this context, and with the present work focusing on amino acids, especially those serving as acrylamide precursors, acrylamide was initially considered a potential food safety marker in the production of plant powders.

**Figure 2:**
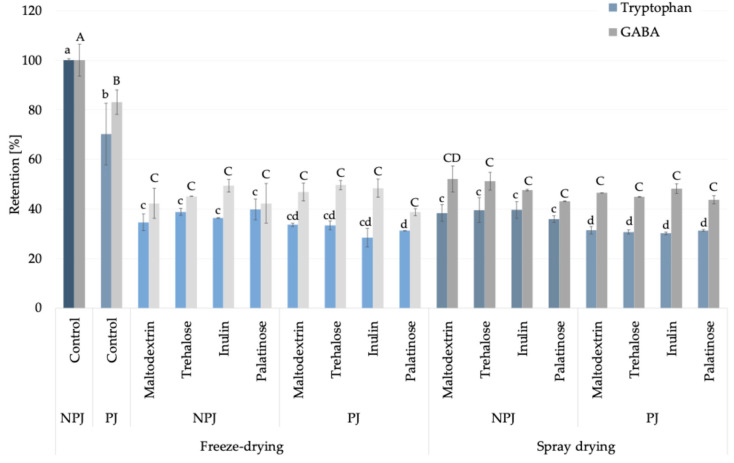
Retention (%) of tryptophan (blue bars) and GABA (γ-aminobutyric acid; grey bars) in *Rosa canina* L. juice during pasteurization without carriers (non-pasteurized juice, NPJ; pasteurized juice, PJ) and after powder production. Lighter shades indicate freeze-drying and darker shades indicate spray drying, both with different carriers. Values are mean ± standard deviation. Different lowercase letters indicate significant differences for tryptophan, and uppercase letters for GABA (Fisher’s LSD post-hoc test, *p* < 0.05).

In this study, based on the amino acid profile identified in *Rosa canina* L. juices and powders (Supplementary Data S1), tryptophan was selected as a representative amino acid indicator, since its pattern provides a reliable marker of thermal processing effects in fruit-based powders. Although histidine was quantitatively predominant, tryptophan was chosen because its moderate abundance, lower reactivity in Maillard-type reactions, and clear sensitivity to heat degradation [19,20] make it more suitable for differentiating pasteurization-induced changes from the stabilization achieved during drying. In freeze-dried, non-pasteurized juice (control), this essential amino acid content reached 48.53 ± 0.31 mg/100 g powder DM, whereas pasteurization caused a significant reduction of approximately 30% (34.06 ± 6.05 mg/100 g powder DM) (Supplementary Data S1). In the case of carrier addition, the observed decrease in tryptophan content was associated with a higher solid fraction introduced by the carriers when compared to non-carrier added samples. Subsequent drying by either freeze-drying or spray drying, with carrier addition, maintained its levels within the range of 13–19 mg/100 g powder DM, indicating that pasteurization rather than atomization was the critical step impacting its stability. The relative retention of tryptophan compared to freeze-dried, non-pasteurized juice powder under different processing conditions is illustrated in Figure 2, further confirming the critical role of pasteurization in determining amino acid stability. Beyond its analytical value, tryptophan is nutritionally relevant as a precursor of serotonin and melatonin, linking its technological behavior with potential health-related functions [21]. For this reason, in the study, tryptophan was highlighted as an exemplary amino acid marker. In addition, among non-essential amino acids, γ-aminobutyric acid (GABA) can be considered alongside tryptophan as a compound of interest (Figure 2), offering insight into amino acids with neuroactive properties [22]. The processing behavior of GABA was similar to that of tryptophan, supporting its suitability as a complementary indicator compound and further emphasizing the nutritional significance of *Rosa canina* L. powders.

#### 2.2.2. Sugar Content in Powders

Four sugars were identified and quantified in the powders. On average, fructose represented 53% of the total sugar content, followed by glucose (44%), sucrose (3%), and maltose (1%), regardless of the type or presence of the carrier and the drying technique applied (Table 2). A comparable trend with fructose being slightly more abundant than glucose was likewise observed in both fresh and dried fruits of *Rosa canina* L. [23].

Overall, the total sugar content in the powders containing carriers was approximately 2.5 times lower than in the control samples (powders without carrier). The lower sugar content observed in powders with carrier addition (20%; *w/w*) was primarily due to the increase in the mass fraction of juice solids resulting from carrier incorporation.

Among the powders obtained from juices with addition of carriers (Table 2) the highest total sugar content was noted in the products containing inulin. On average, this content was approximately 1.8 times higher compared to the mean value noticed in powders with maltodextrin, trehalose, and palatinose. This observation was primarily associated with the higher fructose content, which was approximately 1.9 times greater in inulin-containing samples. This increase can be directly ascribed to the fructose inherently present in the inulin used as a carrier [24]. In the case of this carrier, part of the measured sugars originates from the carrier itself, which contains free fructose, glucose, and sucrose, as well as fructooligosaccharides that are susceptible to partial hydrolysis during processing. Maltose was detected exclusively in the powders with maltodextrin, confirming that the use of such carriers can introduce additional sugars that are either absent or naturally present in minor amounts in the original fruit juice. This highlights the importance of considering carrier composition when interpreting sugar profiles in plant-based powders, as carriers may significantly influence both the nutritional and functional properties of the final products.

In general, the drying technique did not affect the sugars content in analyzed fruit powders.

#### 2.2.3. Free Amino Groups

**Figure 3:**
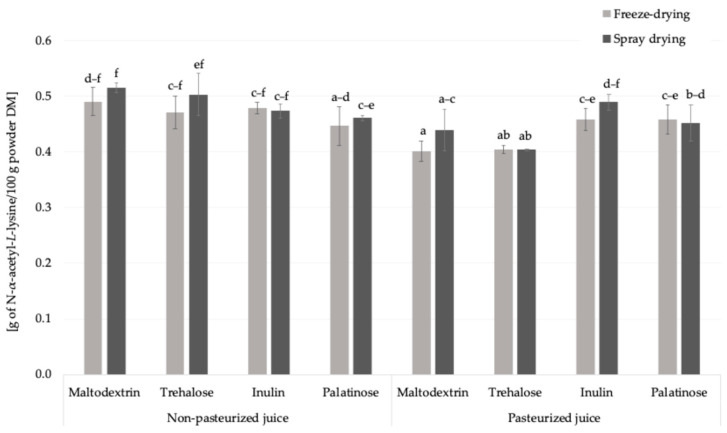
The free amino groups content in *Rosa canina* L. powders; DM—dry matter; a, b, c, d, e, f—different letter with a column indicated a statistically significant difference (Fisher’s LSD post-hoc test, *p* < 0.05).

The free amino groups content was approx. 2.3-times higher in freeze-dried juice than in carrier-added samples which was linked to amino acids content (Table 1, Figure 3). Pasteurization had a noticeable effect on the content of free amino groups. When considering the overall means, irrespective of the carrier type or drying technique, pasteurized samples showed a slightly lower average concentration (0.44 g of N-α-acetyl-*L*-lysine eq./100 g powder DM) compared to non-pasteurized samples (0.48 g of N-α-acetyl-*L*-lysine eq./100 g powder DM). Thermal processes can trigger early Maillard reaction stages and promote interactions of amino acids with various compounds, thereby reducing their availability for derivatization with the OPA reagent.

In this study, the influence of drying technique (spray drying and freeze-drying) and carrier type on the free amino groups content was systematically evaluated for the first time. When considering mean values, a slight effect of the drying technique was observed: samples subjected to freeze-drying showed a marginally higher free amino group content (0.47 g of N-α-acetyl-*L*-lysine eq./100 g powder DM) compared to those obtained by spray drying (0.45 g of N-α-acetyl-*L*-lysine eq./100 g powder DM). Carrier type also affected the level of free amino groups. Among the carriers used, inulin resulted in the highest average content (0.48 g of N-α-acetyl-*L*-lysine eq./100 g powder DM), followed by maltodextrin (0.46 g/100 g powder DM), while trehalose and palatinose showed slightly lower values (both 0.45 g/100 g powder DM) (Figure 3). These results suggest that both the drying conditions and the chemical nature of the carrier matrix may influence the retention or reactivity of free amino compounds in the final powder. Among all the powders analyzed, the lowest content of free amino groups was observed in the sample prepared from pasteurized juice with trehalose as the carrier. This may suggest a greater participation of amino groups in Maillard-type reactions, possibly related to changes in the physicochemical environment that facilitate glycation; however, further investigation is needed to confirm these mechanisms [25]. Taken together, these findings indicate that both processing parameters (pasteurization and drying technique) and carrier type play a decisive role in shaping the availability of free amino groups. Understanding these interactions is essential for adjusting the plant powder production in order to minimize unwanted Maillard-derived changes while preserving nutritional quality.

#### 2.2.4. Fructosamine Content

**Figure 4:**
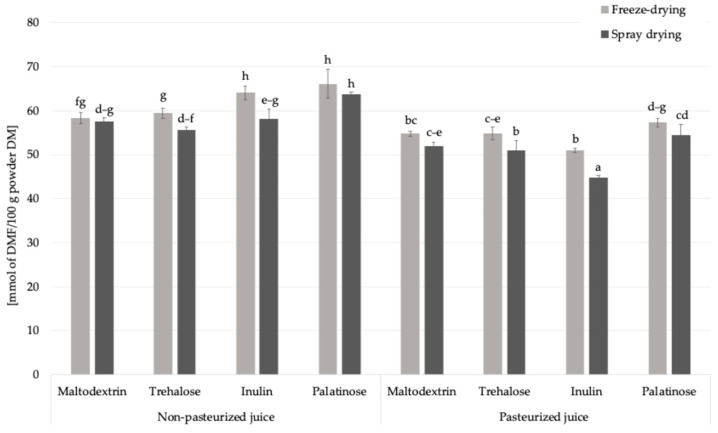
The content of fructosamine in *Rosa canina* L. powders; DM—dry matter; DMF—1-deoxy-1-morpholino-D-fructose; a, b, c, d, e, f, g, h—different letter with a column indicated a statistically significant difference (Fisher’s LSD post-hoc test, *p* < 0.05).

Fructosamine is an early product of the Maillard reaction (Figure 1), formed through the non-enzymatic binding of reducing sugars to the amino groups of proteins or free amino acids. It is commonly used as an indicator of short-term glycation processes in food systems. The content in powdered products obtained from pasteurized and non-pasteurized juice with inulin ranged from 44.8 to 66.2 mmol DMF/100 g powder DM (Figure 4). Pasteurization and the type of carrier significantly influenced the levels of fructosamine in the obtained powders. When assessing the impact of pasteurization, which is a standard treatment applied for fruit juices to ensure microbiological safety and extend shelf life, on fructosamine formation, the control samples were excluded from the analysis to focus solely on the impact of carrier addition. The average fructosamine level in samples produced from non-pasteurized juice was approximately 60.4 mmol DMF/100 g powder DM, while in powders derived from pasteurized juice it decreased to 52.5 mmol DMF/100 g powder DM. This represents a decrease of approximately 13%, suggesting that pasteurization may limit the extent of early Maillard reaction by partially degrading reactive carbonyl and amino compounds or initiating preliminary reaction steps prior to drying, with the reaction potentially progressing into more advanced stages.

This trend was consistent regardless of the carrier type used, although slight variations were observed among individual carbohydrates. The reduction in fructosamine content may also reflect a lower glycation potential due to the thermal pre-treatment of juice, which affects the reactivity of Maillard precursors in the subsequent dehydration step. These results highlight that pasteurization, while widely employed to improve microbial safety and product stability, can also influence the chemical transformations of bioactive components during powder production.

The drying technique also affected the formation of fructosamine in *Rosa canina* L. powders. The average fructosamine content in freeze-dried samples was 58.25 mmol DMF/100 g powder DM, slightly higher than in spray-dried powders, which averaged 54.64 mmol DMF/100 g powder DM. The difference can be explained by the longer duration of freeze-drying compared to spray drying, which results in extended dehydration, which and was previously associated with the formation of specific Maillard reaction products [26]. The type of carrier also influenced fructosamine formation in the obtained powders. The highest average content was observed in samples containing palatinose (60.42 mmol DMF/100 g powder DM), while the lowest was noted for inulin-based powders (54.50 mmol DMF/100 g powder DM). This trend is consistent with the chemical characteristics of the carriers: palatinose, although a reducing disaccharide with relatively slow glycation kinetics, is still capable of participating in Maillard-type reactions under thermal conditions. In contrast, inulin which is a non-reducing polysaccharide with a high molecular weight and limited number of reactive carbonyl groups, likely restricts the progression of glycation, which results in reduced fructosamine formation. However, inulin added to formulations containing fruit juices introduces small amounts of reducing sugars such as glucose and fructose, either inherently present in the juice or released during processing, which could still participate in early Maillard-type reactions.

Among all tested formulations, the highest fructosamine concentration (66.16 mmol DMF/100 g powder DM) (Figure 4) was detected in the non-pasteurized palatinose-based powder, indicating a combined effect of an available reactive sugars and preserved amino groups in the absence of thermal pre-treatment. On the other hand, the lowest value (44.82 mmol DMF/100 g powder DM) was found in the pasteurized inulin-based sample, supporting the view that both pasteurization and the low glycation potential of inulin contribute to limiting the formation of early Maillard reaction products such as fructosamine.

Although a correlation between lysine and arginine levels and the extent of Maillard reaction could be expected, no such relationship was observed in the current study. The values of lysine and arginine are noteworthy, as both amino acids can participate in the non-enzymatic glycation (Maillard reaction), particularly lysine, which is highly reactive in its early stages. However, these results were not directly associated with the measured fructosamine levels or the overall availability of amino groups. As the samples were not hydrolyzed, the amino acid values reflect only their free form. Given that lysine and arginine were present as free amino acids, and considering the fruit-based nature of the matrix, these compounds may have directly participated in early Maillard-type reactions with available sugars.

#### 2.2.5. Acrylamide

For the first time, the presence of acrylamide was evaluated in *Rosa canina* L. powders produced with various carrier additions. Although precursors of acrylamide, such as asparagine and glutamine, were not confirmed in the raw materials used for powder production, acrylamide analysis was undertaken to verify the chemical safety of all formulations, particularly under thermal processing conditions. As illustrated in Figure 1, amino acids can participate in non-enzymatic glycation through the formation of a Schiff base with reducing sugars, leading to early-stage products such as fructosamine. Acrylamide formation, however, is more specific and predominantly originates from asparagine. The absence of both asparagine and acrylamide in the analyzed powders may be explained by several factors: (a) differences in the fruit varieties used, (b) concentrations falling below the detection limits of the analytical method, as suggested by the detectable presence of their acidic counterparts, aspartic acid and glutamic acid, and (c) early conversion of asparagine and glutamine into Maillard reaction intermediates during processing, with their low abundance limiting the formation of measurable derivatives.

Acrylamide was not detected in any of the analyzed *Rosa canina* L. powders, regardless the carrier type used and the drying technique applied for powders production. This absence was confirmed by the consistent and reproducible signals of the internal standard (^13^C_3_-labelled acrylamide), which verified the reliability of the analytical method. In all cases, only the internal standard was detected, while native acrylamide remained below the limit of detection. These findings indicate that the applied processing conditions, including pasteurization, spray drying, and freeze-drying, did not promote acrylamide formation. Consequently, the technological procedures used in the production of *Rosa canina* L. powders can be considered chemically safe with respect to acrylamide generation. Overall, these results suggest that the applicability of acrylamide as a safety indicator in this fruit-based matrix is limited by the low content of its amino acid precursors, underscoring the importance of precursor availability and analytical sensitivity in such evaluations.

#### 2.2.6. Total Phenolic Compounds (Fast Blue BB Method)

**Figure 5:**
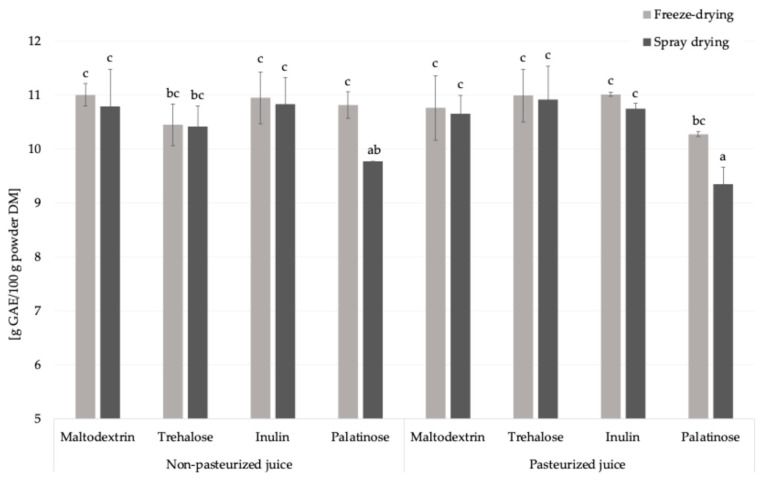
The total phenolics content in *Rosa canina* L. powders; DM—dry matter; GAE—gallic acid equivalent, a, b, c—different letters indicated a statistically significant difference (Fisher’s LSD post-hoc test, *p* < 0.05).

Similar to the amino acid content, the addition of carriers to liquid feed before drying reduced the content of total phenolic compounds, key molecules with antioxidant properties, by approximately 60%, due to the increase in the mass fraction of juice solids after incorporation of carriers (Figure 5) [27]. This observation is consistent with results obtained by the Folin–Ciocalteu method [11]. Pasteurization did not significantly influence the total phenolic compounds content in the resulting powders, regardless of the carrier type or drying technique applied.

Freeze-drying resulted in a significantly higher total phenolics content exclusively in samples containing palatinose, exhibiting an average increase of 10.3% compared to spray drying. This increase was observed using the Fast Blue BB method, whereas no such difference was observed with the Folin–Ciocalteu assay [11]. This discrepancy may be related to the mechanism of the Folin–Ciocalteu method, which detects not only phenolic compounds but also other reducing agents such as ascorbic acid, glucose, fructose, amino acids, proteins, etc., and potential metal chelators [28]. Based on these findings, maltodextrin, trehalose, and inulin provided comparable level of phenolic compounds in analyzed powders. Palatinose was less effective, especially after spray drying.

Although phenolic compounds are not direct participants in the non-enzymatic glycation reaction, they may influence thermal reactivity by modulating redox conditions and interacting with Maillard intermediates. This is particularly relevant in the case of powders produced from *Rosa canina* L., which are naturally rich in polyphenolic compounds such as catechins and phenolic acids [11]. Previous studies have shown that phenolic compounds can covalently bind to proteins and amino acids *via* Maillard-related or oxidative mechanisms, potentially altering the reactivity of amino groups and affecting the progression of thermal reactions [6]. In addition, *Rosa canina* L. is known for its high ascorbic acid content, which may further modulate the redox environment during thermal processing. As a potent antioxidant and reducing agent, ascorbic acid can degrade under heat to form reactive carbonyl species that may interact with amino groups or Maillard intermediates, thereby indirectly influencing browning and the formation of glycation products [29]. Although such specific interactions between phenolic compounds, ascorbic acid, and Maillard intermediates were not evaluated in the current study, they may partially explain some of the observed trends and merit further investigation.

#### 2.2.7. The Principal Component Analysis (PCA)

**Figure 6:**
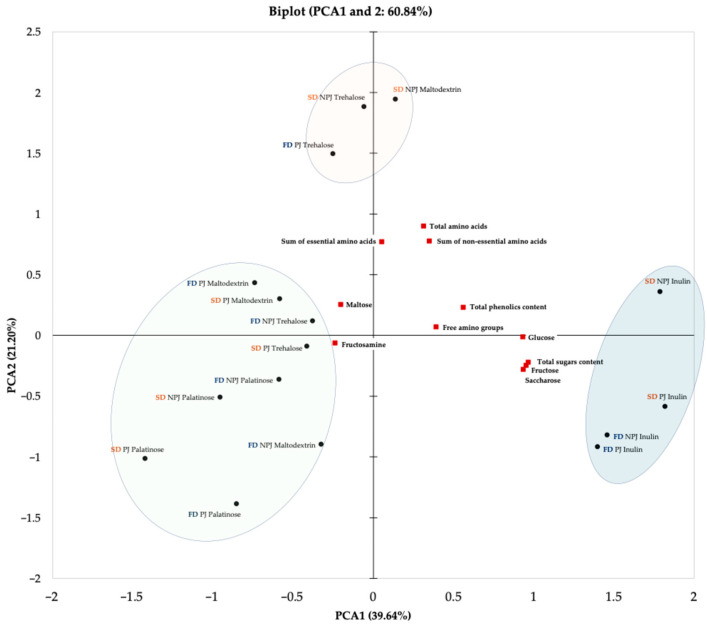
Principal Component Analysis (PCA) biplot showing the two principal components for *Rosa canina* L. juice powders. The PCA illustrates the interrelationships between juice pasteurization, drying methods (freeze-drying and spray drying), and the type of carrier used. PJ—pasteurized juice; NPJ—non-pasteurized juice; FD—freeze-drying (marked in navy); SD—spray drying (marked in orange).

The PCA was performed to explore the relationships between technological variables applied during the production of *Rosa canina* L. juice powders and their chemical properties. The biplot of the first two principal components (PC1 and PC2), which together explain 60.84% of the total variance (PC1: 39.64%; PC2: 21.20%), is presented in Figure 6. The technological variables included in the analysis were juice pasteurization, drying technique and type of carrier (maltodextrin, inulin, trehalose, palatinose). Pasteurization reflects the influence of thermal treatment prior to drying, while the drying technique determines the extent to which bioactive compounds are retained due to differences in processing caused by carrier addition to improve powder obtainment, protect thermolabile compounds, and track the changes linked to formation of process contaminants.

The PCA has shown noticeable clustering patterns based on the analyzed technological variables. The graphical separation into marked areas on the biplot indicates the presence of sample groups with similar chemical profiles and processing conditions. One group included samples produced from both pasteurized and non-pasteurized juice with the addition of inulin, obtained through either freeze-drying or spray drying. These powders showed a positive association with key chemical attributes, including total sugar content, particularly glucose, fructose, and sucrose, as well as total phenolic content and levels of free amino groups. A second group consisted of powders obtained from both pasteurized and non-pasteurized juice with the addition of trehalose, regardless of the drying method, along with powders derived from non-pasteurized juice with maltodextrin. These samples exhibited similar profiles in terms of amino acid content, including both essential and non-essential amino acids. The third group encompassed the remaining samples, particularly those formulated with palatinose. The positioning of these powders on the biplot illustrates how the use of a specific carrier such as palatinose can lead to noticeable changes in the chemical composition of the final product. These groupings reflect the natural clustering of the dataset and emphasize how specific combinations of technological parameters, including juice pretreatment, drying technique, and carrier selection, can influence the chemical properties of *Rosa canina* L. powders in a consistent and predictable manner.

## 3. Materials and Methods

### 3.1. Material

*Rosa canina* L. fruits used in the study were sourced from a local orchard in Kostrzyn Wielkopolski (Poland). After sorting and washing, the fruits were frozen at −18 °C until processing. They were then mechanically cut using a two-step protocol with a high-speed cutter, maintaining a final temperature of 0 °C. The crushed material was enzymatically treated in two 27 kg batches with the addition of water and Pectinex^®^ Ultra SP-L (Novozymes, Bagsværd, Denmark), incubated at 50 °C for 16 h. The resulting products were combined and pressed hydraulically, yielding 45.6 kg of cloudy juice (84.4% yield, 18.28 Bx, pH 3.49). Juice clarification was performed by centrifugation, resulting in 43.2 L of juice and 2 L of sediment.

Half of the juice was pasteurized at 88 °C for 20 s and hot-filled into 5 L bag-in-box containers, while the remaining portion was stored at −18 °C. Both pasteurized and non-pasteurized juices were frozen prior to the preparation of feed solutions for drying [11].

### 3.2. Methods

#### 3.2.1. Drying

The non-pasteurized and pasteurized juices were mixed with 20% (*w/w*): maltodextrin (Pepees S.A., Łomża, Poland), inulin (Beneo-Orafti, Oreye, Belgium), trehalose (Hayashibara Co., Okayama, Japan), and palatinose (PST-N, Beneo-Palatinit GmbH, Mannheim, Germany). Among these, trehalose is classified as a non-reducing sugar, while palatinose, as well as maltodextrin and inulin, may contain or provide reducing sugars capable of reacting with amino acids *via* non-enzymatic glycation, also called the Maillard reaction.

The juice–carrier mixtures were subjected to freeze-drying using a FreeZone system (Labconco Corp., Kansas, MO, USA) for 24 h under reduced pressure (65 Pa) with chamber and shelf temperatures of −60 °C and +24 °C, respectively. Parallel samples were processed by spray drying using a Mini Spray Dryer B-290 (Büchi, Flawil, Switzerland) equipped with a two-fluid nozzle (1.5 mm diameter). The spray drying process was carried out with an inlet temperature set at 130 °C, while the outlet temperature varied between 74 °C and 84 °C, influenced by factors such as the inlet temperature and the type of carrier agent used. The process parameters included a spray volume flow of 414 L/h, a gas flow rate of 26 m^3^/h for samples containing palatinose and 35 m^3^/h for those with other carrier agents, and a liquid feed rate of 4 mL/min. A freeze-dried sample without any carrier addition served as a control. The spray drying and freeze-drying were performed in two technological replications (*n* = 2).

#### 3.2.2. Dry Matter

The dry matter (DM) of powders was determined in duplicate (*n* = 2) using vacuum drying. Samples were dried in a vacuum oven (Vacucell 111 EcoLine, MMM Group, Munich, Germany) connected to a vacuum pump system (MZ 2C NT + AK + EK, Vacuubrand GmbH, Wertheim, Germany) at 80 ± 1 °C under a pressure of 300 Pa for 24 h [27].

#### 3.2.3. Amino Acids Determination by UPLC-PDA-Q/TOF-MS

The extraction was performed in duplicate (*n* = 2) for analysis of amino acids content, following the procedure described by Tkacz et al. [30]. Briefly, approximately 70 mg of each powder sample was extracted with 0.5 mL of 50% methanol (*v/v*) to followed by sonication (15 min) and centrifugation (19,000× *g*, 7 min, 4 °C). The combined the supernatants were submitted to the derivatization by adding 0.2 M boric acid with 5 mM EDTA calcium disodium salt (pH 8.8) and AQC solution (10 mM 6-aminoquinolyl-N-hydroxysuccinimidyl carbamate in acetonitrile) were mixed in a ratio of 1:7:2 (*v/v/v*). The mix was vortexed for 1 min before heating to 55 °C for 10 min and injected to UPLC-PDA-Q/TOF-MS analysis. Obtained results were presented as mg/100 g DM of *Rosa canina* L. powders. 

#### 3.2.4. Sugar Determination by HPLC Coupled with ELSD

Sugar analysis was conducted using a HPLC system (Nexera LC-40, Shimadzu Corp., Kyoto, Japan) equipped with an evaporative light scattering detector (ELSD-LT III). A 2 μL aliquot of each sample was injected onto an ULTRON AF-HILIC-CD (HT) column (2 μm, 100 mm × 3 mm, Shinwa Chemical Industries Ltd., Kyoto, Japan) fitted with a guard column. Chromatographic separation was performed at 45 °C. The mobile phase consisted of 10 mmol/L ammonium acetate in water (solvent A) and acetonitrile (solvent B), with a gradient elution as follows: 15% A and 85% B for 0.5 min; 18% A and 82% B from 3 to 5 min; and 15% A and 85% B from 5.01 to 8 min. The flow rate was maintained at 0.8 mL/min throughout the run. Sugars were identified by comparing retention times to those of reference standards, including glucose, fructose, saccharose and maltose (all purchased from Merck KGaA, Darmstadt, Germany). All measurements were carried out in duplicate (*n* = 2), and the results were expressed as g/100 g DM of powders.

#### 3.2.5. Free Amino Groups (OPA Assay)

For the determination of free amino groups, the extracts were prepared in duplicate (*n* = 2) by mixing approximately 90 mg of powders with 9 mL of distillate water, followed by 30 min of sonication and vortexing (every 10 min) to achieve homogenization. The extracts were used directly for analysis without further treatment. After filtration through Whatman No. 40 paper, the samples were analyzed following the method by Michalska et al. [31], adapted for microplate use. Briefly, 50 µL of sample was mixed with 100 µL of distilled water and 100 µL of OPA reagent (prepared by dissolving 16.4 mg *o*-phthaldialdehyde in 2.5 mL 95% ethanol, adding 25 mL borate buffer (0.1 M, pH 9.5), 400 µL of 10% β-mercaptoethanol, 5 mL of 20% SDS (*w/v*), and diluting to 100 mL with water). The blank contained 50 µL of sample and 200 µL of water. The absorbance was measured at 340 nm a using a Synergy H1 microplate reader (BioTek, Winooski, VT, USA). Results were calculated (*n* = 2) based on a calibration curve with N-α-acetyl-*L*-lysine and expressed as g N-α-acetyl-*L*-lysine/100 g powder DM.

#### 3.2.6. Fructosamine (NBT Assay)

Fructosamine, an early marker of the Maillard reaction, was quantified using the nitro blue tetrazolium (NBT) assay as described by Vlassopoulos et al. [32], with slight modifications [33]. Briefly, NBT was prepared at a final concentration of 0.25 mM in 100 mM sodium carbonate buffer (pH 10.8). For each assay, 25 μL of the sample (*n* = 2) was mixed with 100 μL of the NBT reagent. The reaction kinetics, based on the reduction of NBT and subsequent discoloration, were monitored spectrophotometrically at 530 nm using a microplate reader (PowerWave™ XS, BioTek Instruments, USA) for 20 min at 37 °C. A calibration curve was generated using 1-deoxy-1-morpholino-D-fructose (DMF) as the standard. All measurements were performed in duplicate (*n* = 2), and the results were expressed as mmoles of DMF equivalent per 100 g of powder dry matter (mmol DMF/100 g powder DM).

#### 3.2.7. Acrylamide Content

Acrylamide content was determined using liquid chromatography coupled with tandem mass spectrometry (LC-MS/MS) using a UHPLC Accela liquid chromatograph (Thermo Scientific, San Jose, CA, USA). For extraction, 500 mg of sample mixed with 10 mL of distilled water. Subsequently, 100 μL of an internal standard solution containing ^13^C_3_-labeled acrylamide at a concentration of 1000 ppb was added. The mixture was shaken manually for 30 s, vortexed for 15 s, and then agitated for 60 min using a mechanical shaker at maximum speed. After extraction, the samples were centrifuged at 3600× *g* for 20 min at 10 °C in a refrigerated centrifuge. The supernatant (10 mL) was carefully collected into a clean tube for further purification. Each sample was extracted in duplicate using independently prepared replicates. Solid-phase extraction was performed in two stages using a vacuum manifold system. In the first step, an ISOLUTE^®^ C_18_ cartridge (500 mg/6 mL; Biotage AB, Uppsala, Sweden) was conditioned with 3 mL of methanol followed by two washes with 6 mL of distilled water. The 10 mL extract was passed through the cartridge, and the eluate was collected. In the second stage, this eluate was loaded onto an ISOLUTE^®^ ENV+ cartridge (500 mg/6 mL; Biotage AB, Uppsala, Sweden), previously conditioned with 5 mL of methanol and 5 mL of distilled water. The initial flow-through was discarded, and the column was subsequently rinsed with 4 mL of distilled water. After ensuring that no residual eluate remained in the flow system, acrylamide was eluted with 2 mL of 60% methanol in water. Both the eluate and any remaining solvent from the column were collected and transferred for LC-MS/MS analysis. Chromatographic separation was carried out on an Agilent XDB-C_18_ column (Santa Clara, CA, USA, 4.6 × 150 mm, 5 µm) maintained at 30 °C. The mobile phase consisted of water with 0.1% formic acid (solvent A) and acetonitrile with 0.1% formic acid (solvent B), applied in the following gradient: 0 min—5% B; 10 min—50% B; 15 min—100% B; 17 min—5% B; and held at 5% B until 20 min. The flow rate was set at 0.4 mL/min, and the injection volume was 20 μL. Detection was performed in positive ion mode using selected reaction monitoring (SRM). The MS parameters were as follows: spray voltage, 4500 V; sheath gas pressure, 50; auxiliary gas pressure, 5; capillary temperature, 199 °C. The scan time was set to 0.2 s. Collision energies were set at 9 eV for native acrylamide (transitions: precursor ion *m/z* 72.4, product ion *m/z* 55.5) and 12 eV for the ^13^C_3_-labeled internal standard (transition: precursor ion *m/z* 75.4, product ion *m/z* 58.5). Both tube lens and skimmer voltages were set to 0. All determinations were performed in duplicate (*n* = 2) on independently prepared sample replicates.

#### 3.2.8. Total Phenolics Content (Fast Blue BB Assay)

To avoid interference from non-phenolic antioxidants, reducing agents, and proteins, a more selective method using Fast Blue BB reagent was applied, following Medina [28]. For this analysis, 1 mL of extract was mixed with 100 μL of 0.1% (*v/v)* Fast Blue BB reagent and 100 μL of 5% NaOH. A blank sample, containing 1 mL of extract and 200 μL of deionized water, was prepared to correct for non-phenolic interference. The procedure was carried out in duplicate for each sample (*n* = 2). Samples were manually mixed and incubated in the dark for 1 h. Then, 200 μL of both sample and blank were transferred for absorbance measurement at 420 nm. Results were expressed as g of gallic acid equivalent (GAE) per 100 g sample DM.

#### 3.2.9. Statistical Analysis

The statistical one-way analysis of variance (ANOVA) (Fisher’s LSD post-hoc test) was made to estimate differences between average values (*p* < 0.05) using Statistica 13.1 (Statsoft, Tulsa, OK, USA). Principal Component Analysis (PCA) was employed to reduce the dimensionality of the dataset while preserving the maximum amount of information relevant for interpretation and analysis. The analysis was conducted using the XLSTAT 2022.4.1.1378 software package for statistical and data analysis [34].

## 4. Conclusions

This study provides the first comprehensive characterization of *Rosa canina* L. juice powders produced with different carbohydrate carriers and subjected to various thermal and drying treatments, offering new insights into how formulation and processing influence nutritional values, molecules considered as process contaminants, and components with antioxidant properties that shape the health risk–benefit balance of plant-based processed foods. The results demonstrate that both juice pasteurization and drying techniques significantly affect key molecules, determining nutritional quality, food safety, and antioxidant components. Pasteurization reduced amino acid content and fructosamine levels, while the choice of carrier further modulated these effects. Inulin proved to be the most effective in preserving free amino groups, whereas trehalose in pasteurized powders resulted in the lowest levels. Carrier addition lowered total sugar content in powders but altered sugar profiles through the introduction of exogenous sugars. In this study, tryptophan, representing essential amino acids, and γ-aminobutyric acid (GABA), representing non-essential amino acids, were proposed as indicator compounds for assessing processing effects in *Rosa canina* L. powders. Principal component analysis revealed clear clustering patterns driven by juice treatment, drying technique, and carrier type, grouping the samples according to distinct compositional characteristics for each combination. Importantly, no acrylamide was detected *via* LC-MS/MS, confirming the chemical safety of all tested processing conditions, regardless of the carrier type used or the drying technique applied in powder production.

Overall, these findings highlight the role of processing parameters and carrier selection in modulating the balance between nutritional attributes and potential beneficial or adverse health effects of *Rosa canina* L. juice powders. Juice pasteurization, which is widely applied to ensure the microbiological safety of such products, in combination with carriers differing in their chemical properties, should not be viewed solely in terms of nutritional loss, as it also contributes to lowering fructosamine levels, a molecule linked to food thermal processing. Notably, the use of inulin resulted in the lowest fructosamine content among the carriers tested, while maintaining polyphenolic compounds comparable to those in other powdered products. Assessing the levels of key molecules that serve as indicators of the Maillard reaction, including fructosamine and acrylamide, in combination with nutritional values such as amino acids, sugars, available lysine, and polyphenols, may help determine where a product lies within the health risk–benefit spectrum.

## Figures and Tables

**Table 1:** The amino acids, sugars, free amino groups, fructosamine and total phenolics content in *Rosa canina* L. freeze-dried powders produced from pasteurized and non-pasteurized juice with no carrier addition.

Control Sample	Sum of Amino Acids		Sum of Sugars	Free Amino Groups	Fructosamine Content	Total Phenolics Content
	Essential	Non-Essential				
	[mg/100 g DM]		[g/100 g DM]	[g of N-α-acetyl-*L*-lysine/100 g DM]	[mmol of DMF/100 g DM]	[g of GAE/100 g DM]
Non-pasteurized juice powder	148.82 ± 3.70 b	827.66 ± 35.60 b	16.7 ± 0.18 a	1.08 ± 0.01 a	179.34 ± 0.52 a	26.12 ± 1.08 a
Pasteurized juice powder	107.45 ± 7.43 a	658.07 ± 116.12 a	16.48 ± 0.23 a	1.06 ± 0.05 a	136.26 ± 1.53 b	26.16 ± 0.56 a

DM—dry matter, DMF—1-deoxy-1-morpholino-D-fructose, GAE—gallic acid equivalent; a, b—different letters with the columns indicated statistically significant differences (Fisher’s LSD post-hoc test; *p* < 0.05).

**Table 2:** The content of sugars in *Rosa canina* L. powders (g/100 g powder DM).

	Carrier	Fructose	Glucose	Saccharose	Maltose
		Freeze-drying			
Non-pasteurized juice	-	8.7 ± 0.04 d	8 ± 0.12 h	<LOD	<LOD
Pasteurized juice	-	8.59 ± 0.14 d	7.89 ± 0.09 h	<LOD	<LOD
Non-pasteurized juice	Maltodextrin	2.99 ± 0.1 a	2.94 ± 0.08 ef	<LOD	0.23 ± 0.02 b
	Trehalose	2.94 ± 0.05 a	2.74 ± 0.06 cd	<LOD	<LOD
	Inulin	5.5 ± 0.28 b a	3.18 ± 0 g	1.3 ± 0.1 a	<LOD
	Palatinose	2.9 ± 0.06 a	2.64 ± 0.11 bc	<LOD	<LOD
Pasteurized juice	Maltodextrin	2.78 ± 0.06 a	2.63 ± 0.19 bc	<LOD	0.23 ± 0 b
	Trehalose	2.94 ± 0.04 a	2.73 ± 0.01 cd	<LOD	<LOD
	Inulin	5.55 ± 0.11 b	3.05 ± 0.02 fg	1.27 ± 0.07 a	<LOD
	Palatinose	2.81 ± 0.11 a	2.59 ± 0.16 bc	<LOD	<LOD
		Spray drying			
Non-pasteurized juice	Maltodextrin	2.96 ± 0.02 a	2.88 ± 0.01 d–f	<LOD	0.23 ± 0.03 b
	Trehalose	2.96 ± 0.03 a	2.66 ± 0.15 bc	<LOD	<LOD
	Inulin	5.85 ± 0.36 c	3.14 ± 0.12 g	1.34 ± 0.09 a	<LOD
	Palatinose	2.86 ± 0.04 a	2.53 ± 0.03 b	<LOD	<LOD
Pasteurized juice	Maltodextrin	2.84 ± 0.01 a	2.72 ± 0.01 b–d	<LOD	0.15 ± 0.02 a
	Trehalose	2.86 ± 0.01 a	2.75 ± 0.01 c–e	<LOD	<LOD
	Inulin	6.01 ± 0.03 c	3.2 ± 0.13 g	1.33 ± 0.06 a	<LOD
	Palatinose	2.74 ± 0.02 a	2.24 ± 0.03 a	<LOD	<LOD

DM—dry matter; <LOD—below limit of detection (0.001 g/100 g); a, b, c, d, e, f, g, h—different letter with a column indicated a statistically significant difference (Fisher’s LSD post-hoc test, *p* < 0.05).

## Data Availability

The raw data supporting the conclusions of this article will be made available by the authors on request.

## Supplementary Materials

The following supporting information can be downloaded at: https://www.mdpi.com/article/10.3390/molecules30183805/s1, Data S1: The content of essential and non-essential amino acids quantified in *Rosa canina* L. powders without (control) and with carriers addition (mg/100 g powder DM).
