# Supplementary material for: Impact of Thermal Processing and Carbohydrate Carriers on Amino Acids, Sugars, Phenolic Compounds, and Maillard Reaction Markers Relevant to Nutritional Quality and Safety of Rosa canina L. Juice Powders
Source: Molecules. 2025 Sep 18;30(18):3805. doi: 10.3390/molecules30183805 (PMC12472441; doi:10.3390/molecules30183805)
Supplement: Supplementary file 1 [file molecules-30-03805-s001.zip › molecules-3841253-supplementary.pdf]

molecules

MDPI

Data S1. The content of essential and non-essential amino acids quantified in *Rosa canina* L. powders without (control) and with carriers addition (mg/100 g powder DM).

		Essential amino acids								
	**Carrier**	**Histidine**	**Threonine**	**Lysine**	**Methionine**	**Valine**	**Isoleucine**	**Leucine**	**Phenylalanine**	**Tryptophan**
					Freeze-drying					
Non-pasteurized juice	-	47.04 ± 0.42 b-d	9.72 ± 1.24 e	1.38 ± 0.39 f	2.56 ± 0.14 e	6.19 ± 0.1 c	4.45 ± 0.93 c	16.31 ± 0.37 c	12.63 ± 0.21 f	48.53 ± 0.31 g
Pasteurized juice	-	34.73 ± 3.82 a	7.45 ± 0.07 d	0.79 ± 0.19 e	1.52 ± 0.12 a-c	4.97 ± 0.72 b	3 ± 1.38 b	11.91 ± 1.7 b	9.02 ± 1.4 e	34.06 ± 6.05 f
Non-pasteurized juice	Maltodextrin	43.63 ± 9.75 a-d	4.22 ± 0.69 bc	0.25 ± 0.11 a	1.17 ± 0.2 ab	2.18 ± 0.52 a	1.48 ± 0.25 a	5.47 ± 0.47 a	3.62 ± 0.39 ab	16.8 ± 1.66 a-e
	Trehalose	42.28 ± 1.8 a-d	3.59 ± 0.62 a-c	0.28 ± 0.14 ab	1.36 ± 0.12 a-c	2.14 ± 0.26 a	1.63 ± 0.03 a	5.69 ± 0.44 a	4.38 ± 0.17 b-d	18.83 ± 0.67 de
	Inulin	41.4 ± 6.66 a-d	4.37 ± 0.04 bc	0.52 ± 0.07 b-d	1.27 ± 0.14 a-c	2.1 ± 0.58 a	1.67 ± 0.26 a	5.99 ± 0.19 a	4.73 ± 0.01 cd	17.64 ± 0.1 b-e
	Palatinose	37.32 ± 7.89 ab	4 ± 1.22 a-c	0.4 ± 0.08 a-d	1.24 ± 0.13 a-c	2.32 ± 0.38 a	1.41 ± 0.22 a	6.21 ± 0.86 a	4.67 ± 0.26 cd	19.34 ± 2.03 e
Pasteurized juice	Maltodextrin	51.39 ± 10.89 de	4.01 ± 0.46 a-c	0.38 ± 0.06 a-c	1.1 ± 0.36 ab	2.49 ± 0.06 a	1.74 ± 0.01 a	5.59 ± 0.14 a	4.31 ± 0.18 b-d	16.31 ± 0.34 a-e
	Trehalose	58.73 ± 2.36 e	4.31 ± 0.48 bc	0.41 ± 0.02 a-d	1.35 ± 0.71 a-c	2.46 ± 0.08 a	1.86 ± 0.17 a	6.21 ± 0.21 a	4.09 ± 0.04 a-d	16.17 ± 0.88 a-e
	Inulin	49.79 ± 1.46 c-e	3.21 ± 0.26 ab	0.33 ± 0.07 ab	1.09 ± 0.91 ab	2.39 ± 0.03 a	1.25 ± 0.48 a	5.45 ± 0.26 a	3.28 ± 0.58 a	13.77 ± 1.8 a
	Palatinose	39.77 ± 1.16 a-c	2.83 ± 0.04 a	0.35 ± 0.04 a-c	1.35 ± 0.21 a-c	2.21 ± 0.21 a	1.64 ± 0.03 a	5.39 ± 0.34 a	3.99 ± 0.3 a-c	15.17 ± 0.03 a-d
					Spray drying					
Non-pasteurized juice	Maltodextrin	45.34 ± 2.09 a-d	4.85 ± 0.74 c	0.65 ± 0.09 de	2.44 ± 0.33 de	2.66 ± 0.21 a	1.9 ± 0.02 a	6.39 ± 0.27 a	4.45 ± 0.43 b-d	18.6 ± 1.63 c-e
	Trehalose	47.94 ± 6.4 b-d	4.76 ± 0.56 c	0.42 ± 0.01 a-d	1.69 ± 0.39 a-d	2.63 ± 0.45 a	1.73 ± 0.39 a	6.45 ± 0.28 a	4.76 ± 0.51 cd	19.19 ± 2.45 e
	Inulin	40.38 ± 1.6 a-c	4.54 ± 0.26 bc	0.59 ± 0.03 c-e	2.01 ± 0.56 c-e	2.64 ± 0.05 a	1.95 ± 0.08 a	6.2 ± 0.33 a	4.99 ± 0.15 d	19.23 ± 1.63 e
	Palatinose	41.42 ± 2.35 a-d	4.39 ± 0.17 bc	0.44 ± 0.02 a-d	1.8 ± 0.2 b-e	2.43 ± 0.01 a	1.68 ± 0.15 a	6.06 ± 0.03 a	4.1 ± 0.5 a-d	17.42 ± 0.64 a-e
Pasteurized juice	Maltodextrin	58.65 ± 2.59 e	4.13 ± 0.61 a-c	0.44 ± 0.02 a-d	1.83 ± 0.25 b-e	2.3 ± 0.17 a	1.6 ± 0.18 a	5.38 ± 0.18 a	3.9 ± 0.28 a-c	15.26 ± 0.71 a-d
	Trehalose	46.1 ± 4.09 b-d	3.7 ± 1.06 a-c	0.5 ± 0.08 a-d	1.37 ± 0.03 a-c	2.34 ± 0.11 a	1.59 ± 0.16 a	5.62 ± 0 a	4.19 ± 0.13 a-d	14.91 ± 0.41 a-c
	Inulin	50.38 ± 3.83 c-e	4.24 ± 0.08 bc	0.44 ± 0.09 a-d	1.4 ± 0.28 a-c	2.31 ± 0.3 a	1.22 ± 0.33 a	5.82 ± 0.26 a	4.06 ± 0.12 a-d	14.65 ± 0.25 ab
	Palatinose	46.04 ± 4.83 b-d	4.07 ± 0.89 a-c	0.48 ± 0.01 a-d	0.9 ± 0.39 a	2.34 ± 0.11 a	1.26 ± 0.13 a	5.5 ± 0.26 a	4.12 ± 0.17 a-d	15.2 ± 0.16 a-d

 https://doi.org/10.3390/molecules30183805

		Non-essential amino acids							
		**Homocysteine**	**Tyrosine**	**Alanine**	**Gamma-aminobutyric acid**	**Arginine**	**Glycine**	**Aspartic acid**	**Glutamic acid**
		Freeze-drying							
Non-pasteurized juice		22.12 ± 0.95 H	11.34 ± 0.17 C	24.45 ± 1.39 FG	34.21 ± 3.24 F	563.73 ± 16.92 C	30.86 ± 1.17 E	64.09 ± 3.71 C	72.81 ± 6.93 F
Pasteurized juice		9.65 ± 3.04 FG	8.04 ± 2.59 B	25.35 ± 0.53 G	28.43 ± 1.67 E	467.24 ± 93.67 B	24.92 ± 4.81 D	56.11 ± 12.37 C	31.93 ± 6.62 E
Non-pasteurized juice	Maltodextrin	6.14 ± 0.58 A-E	4.04 ± 0.3 A	16.27 ± 1.11 A-C	14.45 ± 2.07 AB	186.76 ± 24.81 A	12.71 ± 0.27 A-C	20.01 ± 8.43 A	25.44 ± 0.03 C-E
	Trehalose	7.15 ± 1.32 B-F	3.09 ± 1.29 A	21.45 ± 0.78 EF	15.42 ± 0.02 A-D	207.11 ± 12.2 A	14.56 ± 0.08 C	24.53 ± 0.17 AB	23.8 ± 1.85 A-D
	Inulin	7.58 ± 1.13 C-F	4.45 ± 0.54 A	15.53 ± 1.56 AB	16.89 ± 0.86 B-D	198.16 ± 7.53 A	13.45 ± 0.7 A-C	25.74 ± 0.81 AB	28.25 ± 0.92 DE
	Palatinose	8.63 ± 0.89 E-G	4.42 ± 0.59 A	17.75 ± 2.57 B-D	14.45 ± 2.74 AB	206.29 ± 28.4 A	12.75 ± 0.65 A-C	24.14 ± 0.97 AB	22.65 ± 6.51 A-D
Pasteurized juice	Maltodextrin	4.59 ± 0 AB	4.21 ± 0.82 A	16.78 ± 2.94 B-D	16.02 ± 1.25 A-D	202.23 ± 0.04 a	13.72 ± 0.13 BC	28.3 ± 0.22 B	18.11 ± 2.87 AB
	Trehalose	6.23 ± 0.59 A-E	3.7 ± 0.55 A	21.15 ± 2.06 E	16.97 ± 0.66 B-D	215.97 ± 7.23 A	13.04 ± 1.95 A-C	25.49 ± 0.27 AB	17.35 ± 1.34 A
	Inulin	4.88 ± 0.05 A-C	3.28 ± 0.26 A	15.73 ± 1.21 AB	16.56 ± 1.25 B-D	203.95 ± 4.36 A	11.59 ± 1.64 A-C	23.51 ± 2.36 AB	25.05 ± 1.2 B-E
	Palatinose	5.51 ± 1.34 A-C	4.39 ± 0.12 A	15.37 ± 0.03 AB	13.24 ± 0.47 A	198.08 ± 4.31 A	12.2 ± 1.14 A-C	23.93 ± 1.94 AB	22.55 ± 0.95 A-D
		Spray drying							
Non-pasteurized juice	Maltodextrin	10.91 ± 3.31 G	4.12 ± 0.53 A	17.16 ± 0.31 B-D	17.8 ± 1.79 D	218.19 ± 18.8 A	14.17 ± 1.65 C	27.98 ± 3.36 AB	28.35 ± 3.18 DE
	Trehalose	9.46 ± 1.4 FG	4.55 ± 0.17 A	19.31 ± 1.98 C-E	17.51 ± 1.24 CD	221.75 ± 17.14 A	14.74 ± 1.27 C	25.86 ± 0.34 AB	27.7 ± 0.69 DE
	Inulin	9.22 ± 0.48 FG	4.34 ± 0.03 A	19.48 ± 0.79 DE	16.29 ± 0.11 B-D	211.7 ± 4.08 A	14.03 ± 0.5 C	27.5 ± 1.45 AB	31.84 ± 0.99 E
	Trehalose	8.39 ± 0.35 D-G	3.81 ± 0.64 A	16.74 ± 0.93 B-D	14.75 ± 0.05 A-C	197.3 ± 4.5 A	13.11 ± 0.51 A-C	25.83 ± 0.6 AB	26.63 ± 1.65 DE
Pasteurized juice	Maltodextrin	4.83 ± 0.9 A-C	3.4 ± 0.63 A	13.42 ± 1.98 A	15.92 ± 0 A-D	201.3 ± 8.7 A	10.52 ± 1.03 AB	23.65 ± 1.07 AB	18.6 ± 3.76 A-C
	Trehalose	5.58 ± 0.01 A-D	3.59 ± 0.44 A	17.58 ± 0.28 B-D	15.36 ± 0.03 A-D	207.26 ± 3.58 A	12.15 ± 1.56 A-C	23.76 ± 2.16 AB	24.3 ± 1.69 A-D
	Inulin	4.24 ± 1.52 A	3.36 ± 0.37 A	16.62 ± 1.59 B-D	16.46 ± 0.67 B-D	207.35 ± 8.27 A	12.01 ± 0.4 A-C	24.61 ± 0.39 AB	22.43 ± 3.91 A-D
	Palatinose	4.89 ± 0.68 A-C	3.18 ± 0.23 A	15.85 ± 1.28 AB	14.95 ± 0.55 A-D	198.04 ± 7.87 A	10.51 ± 0.13 A	24.52 ± 2.13 AB	22.78 ± 3.51 A-D

DM – dry matter; a,b,c,d,e,f,g – different letter with a column in essential amino acids group indicated a statistically significant difference (Fisher's LSD post-hoc test, $p < 0.05$); A,B,C,D,E,F,G – different letter with a column in essential amino acids group indicated a statistically significant difference (Fisher's LSD post-hoc test, $p < 0.05$
